# Loxapine for Treatment of Patients With Refractory, Chemotherapy-Induced Neuropathic Pain: A Prematurely Terminated Pilot Study Showing Efficacy But Limited Tolerability

**DOI:** 10.3389/fphar.2019.00838

**Published:** 2019-07-25

**Authors:** Sven Schmiedl, David Peters, Oliver Schmalz, Anke Mielke, Tanja Rossmanith, Shirin Diop, Martina Piefke, Petra Thürmann, Achim Schmidtko

**Affiliations:** ^1^Philipp Klee-Institute for Clinical Pharmacology, Helios University Hospital Wuppertal, Wuppertal, Germany; ^2^Department of Clinical Pharmacology, School of Medicine, Faculty of Health, Witten/Herdecke University, Witten, Germany; ^3^Clinic of Internal Medicine I, Division of Oncology and Palliative Care, Helios University Hospital Wuppertal, Wuppertal, Germany; ^4^Clinic for Anesthesiology, Pain Management Unit, Helios University Hospital Wuppertal, Wuppertal, Germany; ^5^Fraunhofer Institute for Molecular Biology and Applied Ecology (IME), branch for Translational Medicine and Pharmacology of the Fraunhofer IME, Frankfurt, Germany; ^6^Neurobiology and Genetics of Behavior, Department of Psychology and Psychotherapy, Centre for Biomedical Education and Research (ZBAF), Witten/Herdecke University, Witten, Germany; ^7^Institute of Pharmacology and Toxicology, Centre for Biomedical Education and Research (ZBAF), School of Medicine, Faculty of Health, Witten/Herdecke University, Witten, Germany; ^8^Institute of Pharmacology and Clinical Pharmacy, College of Pharmacy, Goethe University, Frankfurt, Germany

**Keywords:** loxapine, neuropathic pain, Slack channel, analgesia, tolerability and safety

## Abstract

Neuropathic pain is a debilitating and commonly treatment-refractory condition requiring novel therapeutic options. Accumulating preclinical studies indicate that the potassium channel Slack (K_Na_1.1) contributes to the processing of neuropathic pain, and that Slack activators, when injected into mice, ameliorate pain-related hypersensitivity. However, whether Slack activation might reduce neuropathic pain in humans remains elusive. Here, we evaluated the tolerability and analgesic efficacy of loxapine, a first-generation antipsychotic drug and Slack activator, in neuropathic pain patients. We aimed to treat 12 patients with chronic chemotherapy-induced, treatment-refractory neuropathic pain (pain severity ≥ 4 units on an 11-point numerical rating scale) in a monocentric, open label, proof-of-principle study. Patients received loxapine orally as add-on analgesic in a dose-escalating manner (four treatment episodes for 14 days, daily dose: 20, 30, 40, or 60 mg loxapine) depending on tolerability and analgesic efficacy. Patient-reported outcomes of pain intensity and/or relief were recorded daily. After enrolling four patients, this study was prematurely terminated due to adverse events typically occurring with first-generation antipsychotic drugs that were reported by all patients. In two patients receiving loxapine for at least two treatment episodes, a clinically relevant analgesic effect was found at a daily dose of 20–30 mg of loxapine. Another two patients tolerated loxapine only for a few days. Together, our data further support the hypothesis that Slack activation might be a novel strategy for neuropathic pain therapy. However, loxapine is no valid treatment option for painful polyneuropathy due to profound dopamine and histamine receptor-related side effects.

Clinical Trial Registration: www.ClinicalTrials.gov, identifier NCT02820519.

## Introduction

Neuropathic pain is caused by a lesion or disease of the somatosensory system that can arise from a diverse group of pathological conditions (Baron et al., 2010; Jensen et al., 2011). The prevalence of chronic neuropathic pain in the general population ranges between 7% and 10% (van Hecke et al., 2013) and is even higher in subpopulations such as cancer patients receiving neurotoxic chemotherapeutic agents (Staff et al., 2017). Neuropathic pain has a substantial effect on quality of life, is associated with a high economic burden, and is widely recognized as one of the most difficult pain syndromes to manage (Doth et al., 2010; Attal et al., 2011). In fact, neuropathic pain is generally resistant to over-the-counter analgesics, and opioids display only limited effectiveness. Current first-line treatment recommendations include tricyclic antidepressants, serotonin–noradrenaline reuptake inhibitors, and gabapentinoids (Finnerup et al., 2015). However, less than 35% of patients derive meaningful benefit from all therapeutic approaches available today (Nightingale, 2012; Alles and Smith, 2018). Hence, there is a large unmet clinical need for effective treatment of neuropathic pain (Yekkirala et al., 2017).

Neuropathic lesions are linked to enhanced excitability of sensory neurons. This hyperexcitability is driven by altered expression and activity of ion channels (Waxman and Zamponi, 2014). Potassium (K^+^) channels are the most populous and diverse class of neuronal ion channels, and they are increasingly recognized as potential targets for pain therapy (Tsantoulas and McMahon, 2014; Knezevic et al., 2017). Slack (also referred to as K_Na_1.1, Slo2.2, or Kcnt1) is a K^+^ channel that is highly expressed in nociceptive sensory neurons. Recent preclinical studies suggest that Slack controls the sensory input in chronic pain states (Kaczmarek, 2013; Lu et al., 2015), pointing to Slack activation as a novel strategy for management of neuropathic pain. In a library screen of pharmacologically active compounds, the first-generation antipsychotic drug loxapine was found to activate Slack (Biton et al., 2011). Interestingly, treatment with a low dose of loxapine in mouse models of neuropathic pain considerably reduced the pain behavior in wild-type mice but not in Slack knockout mice, indicating that the loxapine-induced analgesia depends on Slack activation (Lu et al., 2015).

Based on these preclinical data, we hypothesized that loxapine might inhibit neuropathic pain in patients. Loxapine is an approved antipsychotic drug used in psychiatry for over 40 years and available for oral, intramuscular, and inhalative delivery. In addition to the activation of Slack channels, loxapine shows high-affinity antagonism of dopamine receptors (in particular D_2_, D_3_, and D_4_), serotonin receptors (5-HT_2A_ and 5-HT_2C_), histamine receptors (H_1_), and lower-affinity antagonism of other receptors [(Vanelle et al., 1994; Roth et al., 2000; Chakrabarti et al., 2007) and NIMH PDSP website (https://pdsp.unc.edu/databases/kidb.php)]. Although classified as a typical antipsychotic, loxapine has atypical characteristics such as a high 5-HT_2_/D_2_ receptor ratio (Popovic et al., 2015). In psychiatric patients, the usual starting dose of loxapine is 10 mg twice daily, the usual therapeutic and maintenance range is 60–100 mg/day, and the maximum recommended dose is 250 mg/day. Common side effects of loxapine treatment include extrapyramidal symptoms (such as Parkinsonian-like symptoms, akathisia, and tardive dyskinesia), which, however, have been reported to be usually not observed at clinically effective antipsychotic doses (Vanelle et al., 1994; Popovic et al., 2015). Less frequent adverse events include CNS (such as somnolence and drowsiness), anticholinergic, cardiovascular, and gastrointestinal effects. Here, we evaluated the tolerability and analgesic efficacy of orally administered loxapine at a low dose (20–60 mg/day) in a proof-of-principle study in patients with neuropathic pain refractory to standard treatments.

## Materials and Methods

This single-center, dose-escalating, outpatient, open, proof-of-principle study was conducted at the Helios University Hospital Wuppertal, Wuppertal, Germany. The study was registered at clinicaltrials.gov (NCT02820519) and was approved by an independent ethics committee (Witten/Herdecke University; F-183/2014) and the Federal Institute for Drugs and Medical Devices (Bundesinstitut für Arzneimittel und Medizinprodukte, BfArM, Bonn, Germany; EudraCT number: 2014-005440-17) prior to subject screening and enrollment. The study was conducted in conformity with the ethical standards according to the Declaration of Helsinki and Good Clinical Practice guidelines. Participants were compensated for travel expenses and received 50 € for completion of the 8-week trial, but there were no other financial incentives to participate. Written informed consent was obtained from all participants before initiating study-related procedures.

### Inclusion and Exclusion Criteria

We enrolled outpatients (age: ≥18 years, body weight: 50–150 kg) with chemotherapy-induced neuropathic pain (including mixed pain), present on a daily basis for at least 3 months and refractory to at least one analgesic compound, and baseline neuropathic pain intensity ≥4 on an 11-point numerical rating scale (NRS; 0 = no pain; 10 = worst pain imaginable). Exclusion criteria were as follows: Parkinson’s disease, movement disorders (extrapyramidal signs and symptoms) associated with antipsychotics, neuroleptic malignant syndrome, other syndromes associated with antipsychotics, severe hypotension with a syncope in history, glaucoma, urinary retention, epilepsy or other seizure disorders in history, severe dementia, dementia-related psychosis in history, breast cancer in medical history, malignancies with a life expectancy of less than 6 months, other severe and life-threatening diseases, known drug or alcohol abuse, concomitant intake of antipsychotics, dopamine agonists (levodopa, bromocriptine, lisuride, pergolide, ropinirole, cabergoline, pramipexole, or apomorphine), alpha-receptor blocking compounds or compounds with a known potential for QT interval prolongation, pregnancy or lactation period, pre- or perimenopausal females with ineffective contraception, participation in other interventional studies (current or within the last 3 months), and close affiliation with the investigational site.

### Study Design

In this study, it was planned to treat 12 subjects with loxapine in a dose-escalating manner during four 14-day treatment episodes. Loxapine capsules 10 mg (Lannett Company Inc., Philadelphia, USA) were used as investigational medicinal product (IMP) and self-administered on an outpatient basis as add-on treatment to the subject’s usual care (including analgesics). The loxapine dosage for the first episode (days 1–14) was 10 mg b.i.d., dosages for episodes 2, 3, and 4 were defined by taking into account tolerability and analgesic efficacy of the former episode. In case of an acceptable tolerability and if a clinically relevant analgesic efficacy was not reached, loxapine dosage was increased (second episode: 10 mg t.i.d., third episode: 20 mg b.i.d., fourth episode: 20 mg t.i.d). In case of an acceptable tolerability and if a clinically relevant analgesic efficacy was achieved, the dosage of loxapine was not changed. In case of clinically relevant (serious) adverse events [(S)AEs], loxapine dosage was reduced or the treatment was interrupted or stopped (irrespective of the analgesic efficacy).

### Study Procedures

Subjects underwent examinations at the study center during the screening visit (days −6 to −3), at the beginning of each treatment episode (days 1, 15, 29, and 43), at the end of the last treatment episode (day 57), and at the follow-up visit (days 60 to 68).

Baseline neuropathic pain intensity was assessed using an 11-point NRS during the screening visit, at day −2, day −1, day 0, and during hospital visit at day 1 (prior to the first IMP intake), and was defined as the median of these five values. After the first IMP intake, a daily assessment of neuropathic pain using the 11-point NRS, of adverse events, and of analgesic co-medication were conducted by the patients and documented in a diary. A clinically relevant analgesic effect was defined for a particular treatment episode if pain was reduced by at least 30% or two scale units comparing baseline neuropathic pain intensity and the median NRS values of the last 5 days of the respective treatment episode (Frampton and Hughes-Webb, 2011; Haanpää et al., 2011).

Additional procedures in the study center included assessment of the painDETECT questionnaire (Freynhagen et al., 2006a), a patient-reported classification instrument to identify the neuropathic component of pain without clinical examination. The questionnaire includes seven items on sensory symptoms, one item about pain course pattern and one item about the presence of radiating pain. The total score ranges from −1 to 38 points, whereby scores of <13 were considered to indicate unlikely, 13–18 uncertain, and >18 likely presence of neuropathic pain (Baron et al., 2017). Furthermore, we assessed quality of life (QOL) using the 12-item Short Form Health Survey [SF-12v2 (Morfeld et al., 2011)] and anxiety and depression using the Hospital Anxiety and Depression Scale - German version (HADS-D) scale (Hermann-Lingen et al., 2011). These procedures were performed at screening visit, day 1, day 15, day 29, day 43, day 57, and follow-up visit. ECG, safety lab, and vital signs were analyzed at screening visit, day 1, day 15, day 29, day 43, and follow-up visit. Physical examinations were conducted at screening visit and follow-up visit.

### Statistical Methods and Coding of Adverse Events

This pilot study was primarily designed as a safety study evaluating the tolerability of loxapine in non-psychiatric patients. Hence, the primary endpoint was initially defined as the first occurrence of a (serious) adverse event leading to dose reduction or withdrawal of loxapine (“event”). Secondary endpoints were related to tolerability and analgesic efficacy of loxapine. A total number of 12 subjects was initially calculated by a biostatistician. However, the planned statistical analysis was not feasible due to the premature termination of the study and small number of subjects enrolled. Therefore, purely descriptive analysis and graphical presentation were used to elucidate within-subject drug effects and overall findings. All adverse events were coded according to the Medical Dictionary for Regulatory Activities (MedDRA) in its most current version. Severity of adverse events was assessed based on investigator’s decision as “mild”, “moderate”, or “severe”.

## Results

**Figure 1:**
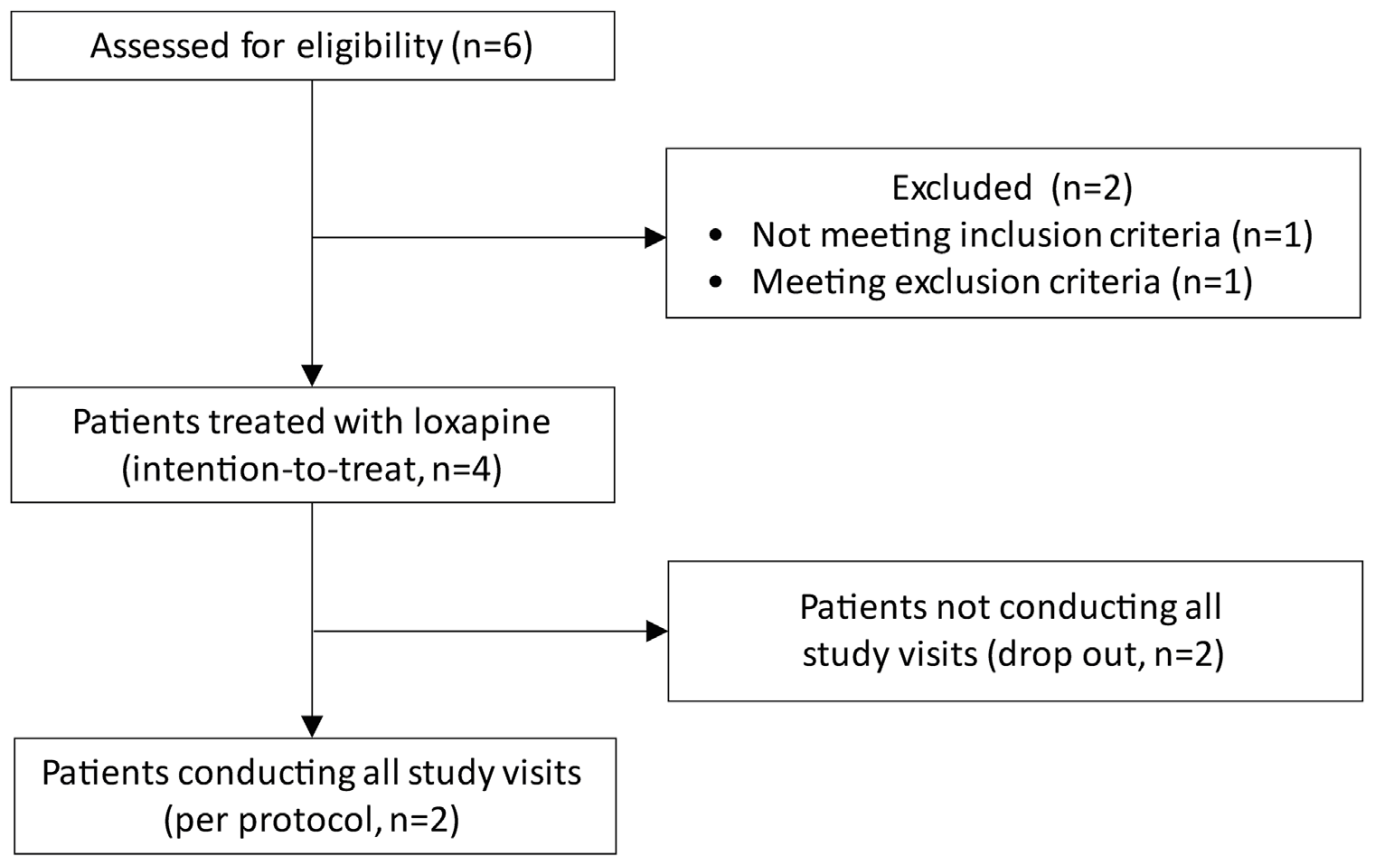
Study flow chart.

Between June 2016 and April 2017, six patients provided informed consent and were screened for eligibility. Two patients could not be included in the study due to pain not fulfilling the inclusion criteria or due to the presence of an exclusion criterion (cachexia). Out of the four subjects receiving IMP (Table 1), two subjects conducted all pre-specified study visits whereas two subjects were not willing to conduct all planned study visits according to the protocol (**Figure 1**). After enrolling these four subjects, the study was prematurely terminated due to a high number of (non-serious) drug-related adverse events and a negative risk–benefit evaluation.

**Table 1:** Baseline characteristics of patients receiving loxapine. Chronic pain with the indicated symptoms and localizations reported at the screening visit was caused by previous anticancer chemotherapy.

Subject	Chemotherapy	Main pain symptoms	Pain localization	Pain duration	NRS at screening	NRS baseline value*
1	5-Fluorouracil, folinic acid, oxaliplatin	Severe: Shooting pain with electrical sensations, prickling, thermal hypersensitivity Moderate: Numbness, allodynia	Hands and feet	6 years	8	8
2	Cisplatin, pemetrexed	Severe: Shooting pain with electrical sensations, thermal hypersensitivity Moderate: Numbness, prickling	Hands, lower legs and feet	1.2 years	5	6
3	Cyclophosphamide, doxorubicin, rituximab, vincristine	Severe: Numbness Moderate: Burning, prickling, thermal hypersensitivity	Legs and feet	4.5 years	7	8
4	Bleomycin, cisplatin, etoposide, ifosfamide	Severe: Shooting pain with electrical sensations, burning Moderate: Numbness, prickling	Hands and feet	14 years	6	5

*Baseline neuropathic pain intensity was assessed using an 11-point NRS during the screening visit, at day −2, day −1, day 0, and during hospital visit at day 1 (prior to the first IMP intake), and was defined as the median of these five values.

### Baseline Characteristics

The four subjects receiving IMP (three males and one female) were aged between 56 and 79 years, had received cytotoxic anticancer drugs suspicious to induce neuropathic pain, and suffered from chronic pain for 1.2–14 years prior to the study (**Table 1**).

### Overall Study Course and Efficacy

**Figure 2:**
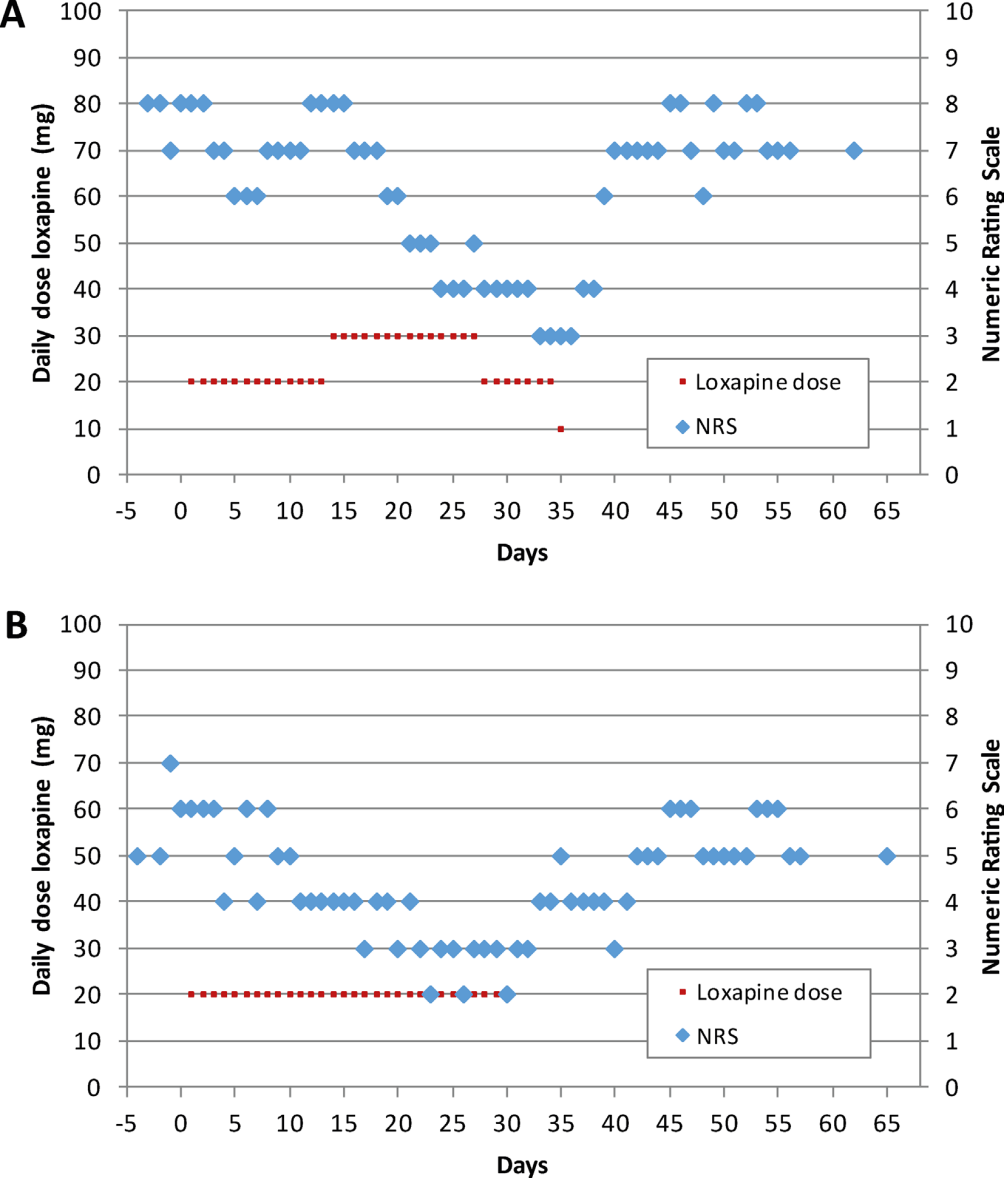
Loxapine dose per day and time course of neuropathic pain as determined by an 11-point numerical rating scale (NRS) for subject #1 **(A)** and #2 **(B)**.

In subject #1, actual analgesic treatment during the screening visit was ibuprofen, which was continued during the study. The painDETECT total score was 21 at day 1, indicating the likely presence of neuropathic pain. The subject tolerated 20 mg/day of loxapine during the first treatment episode. Because pain intensity at day 15 was comparable to baseline values (**Figure 2A**), the loxapine dose was increased to 30 mg/day during the second treatment episode. Notably, thereafter, the pain intensity progressively decreased from 8 to 4 on the 11-point NRS (**Figure 2A**), pointing to analgesic effects of loxapine at a dose of 30 mg/day. However, various adverse events were reported by the patient, especially during the second episode (**Table 2**). Hence, the loxapine dose was reduced to 20 mg/day on day 28. Due to ongoing adverse events (mainly tremor, akathisia, and Parkinsonian gait), the loxapine treatment was prematurely terminated at day 35 after intake of the morning dose (10 mg). After treatment discontinuation, the pain intensity increased, reaching pre-study values after a few days (**Figure 2A**).

**Table 2:** Overview of all reported adverse events during loxapine treatment.

Subject	Adverse events [according to MedDRA hierarchy level “Preferred Term”(PT)]
1	Somnolence, fatigue, muscle rigidity, tremor, akathisia, bradykinesia, Parkinsonian gait, oromandibular dystonia, trismus, decreased activity, memory impairment, vision blurred, spinal pain, hypertension, urinary retention, agitation*, fear*
2	Somnolence, tremor, Parkinsonian gait, restlessness, arthralgia, pruritus generalized, pleural mesothelioma malignant recurrent, initial insomnia*
3	Somnolence, dizziness, nausea
4	Somnolence, burning sensation, allodynia

*Drug withdrawal events after discontinuation of loxapine.

Subject #2 was treated with pregabalin at the screening visit and during the study. Neuropathic pain was likely existent at day 1 as suggested by a PainDETECT total score of 20. After the intake of loxapine 20 mg/day during the first treatment episode, the subject reported clinically relevant analgesia at day 15 (NRS value: 4; **Figure 2B**). Therefore, the loxapine dose was not changed during the second treatment episode. At day 29, when the analgesic effect was still present, the loxapine treatment was prematurely stopped due to adverse events (mainly tremor, Parkinsonian gait, and restlessness). Within the next few days, the pain intensity increased (**Figure 2B**).

In subject #3, actual analgesic treatment during the screening visit and the study was ibuprofen. The presence of neuropathic pain was uncertain at day 1 according to a PainDETECT total score of 18. The subject stopped taking loxapine 20 mg/day after 4 days due to adverse events (mainly somnolence and dizziness). No changes in pain scores were found between baseline and day 4 (NRS: 8, respectively).

In subject #4, analgesic treatment at the screening visit included ibuprofen, morphine, pregabalin, and quetiapine, an atypical antipsychotic drug with potentially analgesic properties (Jensen et al., 2008; Jimenez et al., 2018). Quetiapine was discontinued the day before the first intake of loxapine; all other drugs were pursued during the study. The PainDETECT total score was 18 at day 1, indicating uncertain neuropathic pain. The subject stopped taking loxapine 20 mg/day after 3 days due to adverse events (mainly somnolence and allodynia). Furthermore, the subject reported increased pain intensity (NRS values at baseline: 5, at day 2: 8, at day 3: 7). After re-initiating quetiapine, the pain improved and reached pre-study intensity.

### Adverse Events

Adverse events occurred in all subjects receiving loxapine (**Table 2**). In addition to somnolence, which was reported by all subjects, extrapyramidal and anticholinergic side effects were of particular relevance in the two subjects receiving loxapine for at least 4 weeks. Moreover, in these two subjects, withdrawal symptoms (such as agitation, fear and initial insomnia) occurred after stopping loxapine. Apart from a relapse of a malignant disease, which was diagnosed in subject #2 25 days after loxapine discontinuation and considered not to be related to loxapine intake, all adverse events recovered within a few days after loxapine discontinuation. No serious adverse event occurred in this study. No clinically significant changes were revealed by ECG, safety laboratory measurements, or physical examination.

### Quality of Life, Anxiety, and Depression

The SF-12v2 and HADS-D results for the two patients receiving loxapine for at least two treatment episodes are presented in **Table 3**. In subject #1, SF-12v2 data show only small changes in mental health during day 1 to day 57. In contrast, diminished mental health was found in subject #2 during loxapine intake (day 29), which improved after loxapine discontinuation. In both patients, the SF-12v2 physical health score deteriorated during loxapine intake and improved after stopping loxapine. In the HADS-D anxiety score, only small changes were found in subject #1 during the whole study. In contrast, an increased level of anxiety occurred in subject #2 at day 29, which diminished after the withdrawal of loxapine and was then followed by a transient increase. With regard to the HADS-D depression score, changes were similar to those seen for each patient’s anxiety data.

**Table 3:** 12-item Short Form Health Survey (SF-12v2) and HADS-D scores.

	SV	Day 1	Day 15	Day 29	Day 43	Day 57	FU
SF-12v2/mental health							
Subject #1	54.2	52.7	57.4	59.2	56.78	60.4	56.5
Subject #2	64.2	58.7	51.7	36.7	63.2	57.6	41.0
SF-12v2/physical health							
Subject #1	32.0	42.5	39.1	30.9	38.4	39.3	44.4
Subject #2	37.4	38.4	30.0	32.3	26.9	36.9	33.5
HADS-D/anxiety							
Subject #1	6	4	5	3	1	5	4
Subject #2	3	2	3	11	4	12	7
HADS-D/depression							
Subject #1	3	1	2	2	1	2	1
Subject #2	2	1	1	7	1	4	2

SV, screening visit; FU, follow-up visit.

## Discussion

In this pilot study, we assessed the effects of loxapine, an antipsychotic drug with off-target activity on Slack channels, in patients suffering from refractory neuropathic pain after chemotherapy. The study was prematurely terminated because antipsychotic-related adverse events occurred in all subjects. Hence, we conclude that loxapine is no valid treatment option for this type of painful neuropathy. Nevertheless, the analgesic effects of loxapine observed in this study in combination with results from preclinical studies further support the hypothesis that Slack activation might be a novel strategy for neuropathic pain therapy in the future if novel compounds with improved pharmacological profile are available.

Based on reports that Parkinsonian-like symptoms are not usually observed at clinically effective antipsychotic doses (Vanelle et al., 1994; Popovic et al., 2015) and that treatment with a low dose of loxapine in mice (0.175 mg/kg intraperitoneal) reduced the neuropathic pain behavior in a Slack-dependent manner (Lu et al., 2015), we chose a dose range of 20–60 mg loxapine per day in our study. However, common side effects of oral loxapine were unexpectedly reported by all subjects. In comparison to our study, side effects were substantially less frequent in patients receiving loxapine for treating acute or chronic schizophrenia despite the usage of higher doses. For example, whereas somnolence/sedation occurred in all four subjects in our study, it was present in only 28% of patients with schizophrenia (Heel et al., 1978). Similarly, extrapyramidal signs/symptoms were found in 39% of schizophrenic patients, whereas in our study, the two patients receiving loxapine for a longer period were both affected (Heel et al., 1978). About the reasons for the high incidence and the intensity of adverse events found in our study, we can only speculate. One reason might be the fact that the enrolled patients were elderly and multimorbid. In general, the changes in physiology and homeostasis associated with multimorbidity and increasing age increase the risk of drug toxicity (Wilder-Smith, 1998), leading to a higher vulnerability for developing adverse drug reactions in elderly patients (Lampela et al., 2015). These factors may also contribute to the withdrawal symptoms that occurred after loxapine discontinuation in the two patients receiving loxapine for at least 4 weeks (Azermai et al., 2013; Declercq et al., 2013).

The SF-12v2 scores suggest that the analgesic effect of loxapine in subject #1 and #2 was irrespective of mental and physical health problems that may at least in part have resulted from loxapine intake. The HADS-D scores indicate that subject #1 was emotionally rather stable, while subject #2 was emotionally instable. In particular, the anxiety data (and less prominent also the depression data) of subject #2 suggest that other life events, and not loxapine, induced changes in emotional states. It also needs to be taken into consideration that subject #2 was treated with pregabalin, which has anxiolytic effects. Together, the SF-12v2 and HADS-D data indicate that subject #1 and #2 strongly differed from each other with respect to mental and emotional health. It can thus be concluded that loxapine had analgesic effects in two subjects with highly differential characteristics in psychological health.

In subject #1 and #2, the analgesic effect of loxapine started slowly reaching a clinically relevant analgesia after 2–3 weeks. This delayed onset of action may be associated with the multifactorial genesis of chemotherapy-induced neuropathic pain (Starobova and Vetter, 2017) and was also observed in other clinical trials using, for example, pregabalin (Saif et al., 2010) or duloxetine (Smith et al., 2013). The fact that subject #4 reported increased pain at 2–3 days after the treatment onset with loxapine might therefore, although speculative, be related to the discontinuation of quetiapine prior to the first loxapine intake. In fact, the atypical antipsychotic drug quetiapine differs from other atypical and typical antipsychotic drugs by its antidepressant activity, which seems to be mediated by its metabolite *N*-desalkylquetiapine through partial 5-HT_1A_ agonism and inhibition of the norepinephrine reuptake transporter (Jensen et al., 2008). The latter mechanism might also mediate analgesic effects that have been reported in clinical trials (Jimenez et al., 2018).

Our pilot study has important limitations that impair the generalizability of the results. These include the small sample size, the short study duration, its exploratory nature, the open-label design, the lack of a placebo control, and the high rate of dropouts caused by adverse events. Interestingly, a substantial but somewhat lower dropout rate (approximately 20%) was found in a dose-escalating study evaluating pregabalin in patients with neuropathic pain (Freynhagen et al., 2006b). As stated before, our patients are probably suffering from a higher number of clinically relevant comorbidities explaining our dropout rate (2 out of 4 patients, i.e., 50%) at least to some extent. Furthermore, due to the pilot character of our study, “treatment refractory” was defined as refractory to any analgesic, i.e., not necessarily including compounds used as a first-line option for treating neuropathic pain. Because of these limitations, no definitive conclusions or generalizations can be made. However, the data are consistent with a possible analgesic effect of loxapine in patients suffering from neuropathic pain, thereby providing sufficient evidence to warrant further investigations. In particular, the development of new drugs with Slack-activating properties but improved pharmacological profiles compared to loxapine might be considered.

## Data Availability

The datasets generated for this study are available on request to the corresponding author.

## Ethics Statement

This single-center, dose-escalating, out-patient, open, proof-of-principle study was conducted at the Helios University Hospital Wuppertal, Wuppertal, Germany. The study was registered at clinicaltrials.gov (NCT02820519) and was approved by the institutional ethics committee of Witten/Herdecke University (F-183/2014) and the Federal Institute for Drugs and Medical Devices (Bundesinstitut fuer Arzneimittel und Medizinprodukte, BfArM, Bonn, Germany; EudraCT number: 2014-005440-17). The study was conducted according to the Declaration of Helsinki and Good Clinical Practice guidelines. Participants were compensated for travel expenses and received 50 € for completion of the 8-week trial, but there were no other financial incentives to participate. Written informed consent was obtained from all participants before initiating study-related procedures.

## Author Contributions

SS and AS designed the study and wrote the manuscript. All authors contributed substantially to acquisition, analysis, and interpretation of data; discussed the results; and commented on the manuscript.

## Funding

This study was supported by an internal research funding of the Witten/Herdecke University, Witten, Germany. The investigational compound was provided in parts free of charge by Lannett Company Inc., Philadelphia, USA.

## Conflict of Interest Statement

The authors declare that the research was conducted in the absence of any commercial or financial relationships that could be construed as a potential conflict of interest.
